# The Impact of Stress on Innovative Work Behavior among Medical Healthcare Professionals

**DOI:** 10.3390/bs12090340

**Published:** 2022-09-16

**Authors:** Amna Anjum, Yan Zhao

**Affiliations:** School of Management, Shanghai University, Shanghai 200444, China

**Keywords:** eustress, distress, health, innovative behavior, supervisor support

## Abstract

Background: For health systems, a fundamental challenge is adapting to changes in the patterns of health services that require technological and scientific innovations. The pace of multiple and interconnected challenges mounts extra stress on medical healthcare professionals and reduces their innovative capabilities, especially in low- and middle-income countries. To enhance the innovative capability of medical healthcare professionals under stress, the study seeks any possible correlation between stress and innovation. For that purpose, we sought to investigate the effects of stress on the innovative work behavior of employees and examine the mediating effect of health and moderating effect of supervisor support. Materials and Methods: 350 medical healthcare professionals were surveyed in different hospitals in Lahore through a survey regarding stress, health, innovative work behavior, and supervisor support with a final response rate of 89%. SPSS and AMOS were used for the analysis of the data and the investigation of the combined effects of the model. Exploratory (EFA) and Confirmatory Factor Analysis (CFA) were conducted to ensure the convergent and discriminant validity of the factors, while mediation analysis was done to check the mediating role of health. Results: It has been observed that there is partial mediation of health between eustress and innovative work behavior whereas supervisor support does not moderate between eustress and health. Furthermore, the results indicate that distress is negatively associated with innovative behavior. In addition, health fully mediates between distress and innovative work behavior. If distress increases negative effects on health, then supervisor support reduces the negative impact of distress on health. Furthermore, supervisor support also reduces the negative impact of health on innovative work behavior. Conclusion: Our study outlines a hypothetical alternative situation that explains how the two emotions of eustress and distress are brought into play in the innovative work behavior of the employees. In addition, supervisors play an important role in influencing the sustainable innovation work behavior of their staff members.

## 1. Introduction

The context in which innovation is measured mostly pertains to the inputs, processes, and outputs of an organization. However, innovation is largely determined by an employee’s innovative behavior at work. The level of innovation that an employee is capable of may be improved in the workplace by improving his or her overall innovative work behavior. This may result in a reduction of errors that relates to work. There is no doubt that healthcare professionals are the pillars of any nation’s health sector because they are the ones who make this sector successful and thrive [1]. Medical healthcare professionals (MHCPs) play an imperative role in the continued development of health services, which is closely related to continuous innovation. Due to this development, employees in the health sector are surrounded by an environment that encourages them to behave creatively at work. A variety of studies have been conducted to point out the problems, stress sources, and sources of tension amongst MHCPs working in developing countries [2]. For an employee to be innovative, he or she must work in an environment in which there is no stress. Even though there is a considerable amount of research on the direct effects of stress on health, there is very little on the mediating role of health in this process.

There is no doubt that work stress can have a significant impact on the health of healthcare workers, as well as their innovation at work; therefore, it is urgent for this population to improve their innovative work behavior while controlling stress at work. The main concern when it comes to stress is its adverse impact on the health of medical healthcare workers, as well as their ability to innovate.

As a general rule, it is believed that a certain amount of stress that can be handled both mentally and physically is referred to as good stress (or “eustress”), while a certain amount of stress that cannot be handled either mentally or physically is considered bad stress (sometimes called distress). Eustress has not been studied extensively in the literature. There are almost 148 items found in a search for the term “eustress” in psychology, psychiatry, social science, and interdisciplinary and behavioral sciences in the Web of Science core collection, while there are 111,945 items found in a search for distress, till 13 September 2022. It is believed that distress is the result of high demands in a job coupled with a low level of control over the situation. These factors can contribute to stress. As a professional, no matter where you work, it is imperative to pay attention to your stress levels. This will ensure you will be able to cope with the side effects of stress no matter what kind of environment you work in.

Distress and eustress have a very slight difference in their meanings. If you are able to bear the level of stress you can tolerate, which puts an extra sense of responsibility on you, you may be able to have a more sustainable and innovative work attitude. On the other hand, the moment you realize that the amount of stress you are under now is out of your control, it leads to distress and disrupts your ability to work in a creative way [3]. As far as distress and eustress are concerned, there is a gray area between them, where they are different from each other. One of the oldest and most important ideas in stress management explains this difference with the “Inverted-U” relationship between distress and eustress, which is one of the earliest and most important concepts in stress management [4]. As far as the effects of stress are concerned, studies have not distinguished between eustress (stress that contributes to career development, such as shift positions, job responsibilities, and workload) and distress (stress that is perceived as unbearable, such as burdensome work policies, conflicts with others, and job insecurity). The relationship between stress and health has been the subject of a growing number of studies over the last few years. Despite the fact that it is obvious that when a medical healthcare worker is in poor health and that it is not possible to engage in innovative work behavior because of stress, researchers have primarily focused on the direct effects of stress on health and neglected the possible mediating effects of health.

Furthermore, a lot of literature is available on the impact of stress on health with different stressors (workload, job insecurity, long working hours, low income, role ambiguity, job dissatisfaction, poor performance, poor peer relations, fewer opportunities for career growth, unsound organizational policies, and practices, poor physical environment). There is, however, some evidence that suggests a stressed mind and an innovative mind do not necessarily get along very well with each other. As a matter of fact, some studies have found that stress negatively impacts the ability to innovate [5,6,7,8]. Taking into consideration these findings, as well as numerous other studies that have documented the detrimental effects of stress on employee performance under stressful conditions [9], raises a theoretically and practically relevant question: how and under what conditions can innovative performance be enabled in stressful conditions?

To answer this question, we have added supervisor support as a moderator in our study. Supervisor support increases organizational support, as stress is inversely proportional to organizational support [10]: employees’ perception that their supervisors value their contribution and care about their well-being may lead them to believe that organizations will also favor them because they are agents of the organization. Based on the integration of these theoretical perspectives, we develop and test a first-stage mediation model that identifies the following mediating processes: (1) health is a mediating process underlying a negative/positive relationship between eustress and innovation in the organization, and (2) health is a mediating process underlying a negative/positive relationship between distress and innovation in the workplace. During the second phase of this mediated relationship, supervisor support plays an even greater role.

The present study intended to address these important research gaps by drawing upon the theories of psychology and management of stress developed by Lazarus and Folkman [11] as well as organizational support theory [12,13,14] as the theoretical framework for this study. Folkman and Lazarus [15] defined two foremost classifications of managing the stress response, whereby a person could endeavor to manage stress by consuming a new kind of rational problem-solving approach (problem-focused managing of stress) or orientation of emotion approach (emotion-focused managing of stress), each of which is appropriate for eustress. Furthermore, according to Lazarus and Folkman’s theory, the inability to manage stress successfully (e.g., from larger demands or shortage of assets or professional experience) is likely to lead to stress and negative medical results [15].

Supervisor support is a key factor that can compensate for the effects of stress on health and on innovative work behavior. Therefore, using our study as an example, we provide a new theoretical lens for understanding the processes and boundary conditions involved in understanding how stress impacts the innovativeness of employees. In addition, it has been shown that stressful work conditions are an important impediment. The research approach to stress that we are taking discloses the role of supervisor support as an individual resource that is expected to uphold the hope of the employees regardless of stress, thus maintaining innovative work behavior despite the stress at work. As a result of our study examining the moderating effects of supervisor support, we are in the process of extending our knowledge of what this individual characteristic can contribute to the relationship of the studied variables.

## 2. Conceptual Framework of Innovative Work Behavior

### 2.1. Eustress and Distress

Stress can never always be a negative experience according to Sullivan [16]. Working under pressure could lead to both positive and negative stress (eustress and distress, respectively). There are a number of demands associated with high levels of stress, such as high responsibilities, a high level of job scope, a high level of workload, and time pressures that have been considered obstacles to achieving the needed outcome [17,18]. It has been shown that the employee’s performance objectives are stretched when faced with eustress, but they are still feasible to reach through hard work, and with a reasonable degree of risk-taking. As opposed to that, distress is the result of demands that seem threatening. It is common for employees to feel that they have no control over their jobs when they are faced with distress. There is an important link between individuals’ working attitudes and their psychological and physical health [19]. Untreated working stress can negatively affect individuals’ psychological health and ability to perform innovative jobs within the organization. Eustress, on the other hand, is generally regarded as a positive stress factor on an individual, in which a work requirement is viewed as an opportunity to enhance self-development and achieve goals, such as a manageable workload that requires little hard work. The eustress effect incites positive emotions, increases performance and motivation, improves innovative attitude, and allows for an increase in the quality of work, which leads to an improvement in innovative behavior. The above discussion leads to the following hypotheses:

**Hypothesis** **1.** *There is a significant relationship between distress and innovative work behavior.*

**Hypothesis** **2.** *There is a significant positive relationship between eustress and innovative work behavior.*

### 2.2. Health

In a lot of ways, having a job makes a person feel better and enables them to have a better outlook on life overall. However, there are significant challenges as well that outweigh any possible benefits and even pose a risk to one’s health. In the modern world, work-related distress has become a growing problem that not only affects the employee’s health but also influences their innovative work behavior through their health. The National Institute for Occupational Safety and Health of the United States defines stress as those harmful physical and emotional reactions triggered by the demands of the job that are out of proportion to the individual’s abilities, resources, or needs. As a result, stress can cause poor physical and mental health, as well as a variety of injuries. When employees are under stress at work, they are more likely to engage in unhealthy behaviors. These harmful health effects ultimately reduce the likelihood of employees engaging in innovative behavior at work.

Despite this, Edwards and Cooper [20], in the most extensive review of the subject of eustress, suggest that eustress can either improve health by directly modifying hormonal and biochemical substances or indirectly by facilitating effort and abilities directed towards effectively coping with existing distress. It was found that a variety of sources, including anecdotal evidence, laboratory experiments, and studies of positive life events and the workplace were used to review the findings. Manageable stress increases alertness and also influences the work behavior of employees.

**Hypothesis** **3.** *The relationship between distress and innovative work behavior is mediated by health.*

**Hypothesis** **4.** *The relationship between eustress and innovative work behavior is mediated by health.*

### 2.3. Supervisor Support

Support from supervisors is based on the theory of organizational support theory [13,14]. It makes it clear that supervisors aim to build strong and long-lasting social bonds among their co-workers, so as to increase the collaboration and responsiveness among them and to ensure the ultimate success of their organization. Supervisor support can be defined as the level to which employees perceive their supervisors as being ready to assist them with work-related issues or as a means of assisting them with the completion of their assigned work or targets. Employees can share their knowledge and experience with their co-workers with supervisor support. In the context of MHCPs working in Pakistan, supervisor support can also play a very important role. In some developing countries, the Doctors Association is always protesting to force the government to accept and compensate them for their legal rights [21,22]. If the employees would have supervisor support, their legal demands could be put forward to the concerned authorities. It would help to create a stress-free working environment.

**Figure 1:**
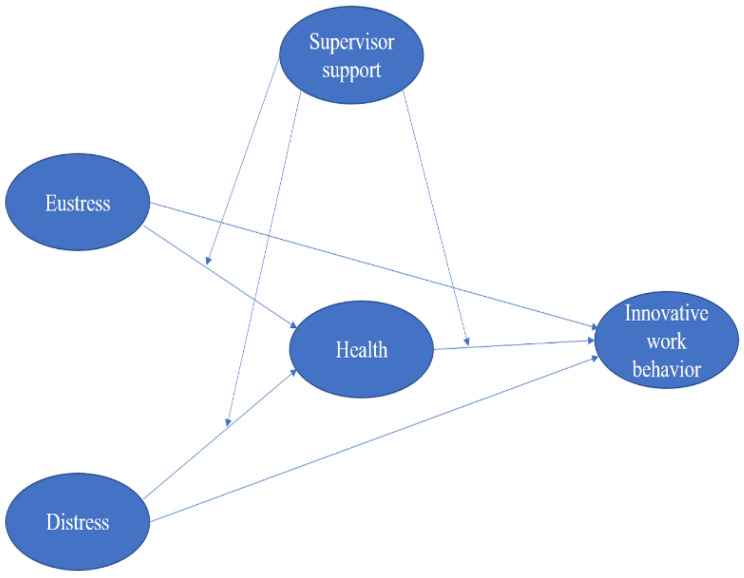
Proposed model of the study.

In order to prevent stress in the organization, identifying the potential sources of stress within an organization is the first step in addressing these issues [2]. Supervisors or managers who are able to effectively reduce job stress can improve the mental and physical health of their employees. This type of supervisory/managerial intervention could be considered a primary intervention that involves proactive preventative measures to reduce stress by removing or reducing potential stressors. As part of this level of intervention, the sources of physical and psychosocial stress are targeted. We propose that the primary interventions for reducing stress could be ensuring that the employees have respite time, allowing them to take naps when they need them, and encouraging full participation in decision-making and planning, all of which will let them feel important in the organization. It is possible for supervisors to increase the time and resources available for the completion of specific job tasks, which could prove beneficial in enhancing their innovative work behaviors. A supervisor’s role is to match job descriptions with employees’ skills and qualifications so that they can have better innovative behavior, which is a very important factor. In order to motivate employees, supervisors will have to amend the policies by creating clear promotions and rewards pathways. Employees will work hard and will strive to do their best to earn their rewards and promotions so that they can eventually develop innovative behavior in the employee despite their bad health. There are a number of things supervisors can do to alleviate this worrisome concern in order to create a healthier, safer, and more productive workplace. A detailed model has been given in Figure 1.

**Hypothesis** **5.** *Supervisor support moderates the relationship between distress and health.*

**Hypothesis** **6.** *Supervisor support moderates the relationship between eustress and health.*

**Hypothesis** **7.** *Supervisor support moderates the relationship between health and innovative work behavior.*

## 3. Measures

We used only English scales for the measurement of variables. Cronbach’s alpha value was used to verify the internal consistency of the scale. Regarding job stress, we divided it into eustress and distress when developing the scale, which included 11 items, with stress comprising a 5-item scale and distress comprising a 6-item scale. Using Cavanaugh et al. [23], we divided it into eustress and distress. An SF-8 survey form was used to assess the respondents’ health. Short-Form Health Survey (SF-8) is a current, vigorous, 8-item tool for assessing health. It deals with the eight health domains (sentimental, limited role, physical pain, social, physiological, physical role, vitality, and overall health) and it is best suited for observing population health at large scale. An innovative work behaviour questionnaire which was based on 21 items with a Cronbach’s α of 0.91 [24] was used to measure the innovative attitude of the employees. Three separate components were measured with regard to the innovative work behavior of an employee: the innovation system (10 items), the competitors and technology (7 items), and new services (4 items). A survey of supervisor support was used developed by Robin Huntington, Steven Hutchison, and Debora Sowa [25] based on eight items, three of which relate to whether the supervisor is willing to help their employee, two of which relate to how valuable an employee’s suggestions are to the supervisor, and the last two relate to the supervisor’s concern for their employees’ health. Using a Likert scale, the responses were categorized as either “strongly disagree” or “strongly agree”, with 1 emphasizing strong disagreement and 5 emphasizing strong agreement.

## 4. Methods

Physicians working across wards/departments of selected public/private hospitals (Fatima Memorial Hospital Lahore, Shaukat Khanam Hospital Lahore, Hamid Latif Hospital Lahore, Punjab Institute of Mental Health Lahore) in Pakistan were surveyed following ethics approval from the hospital committees. All subjects gave their informed consent for inclusion before they participated in the study. The study was conducted in accordance with the Declaration of Helsinki, and the protocol was approved by the Ethics Committee of the Punjab Institute of Mental Health (PIMH). An Institutional Review Board (IRB) reviewed and approved the research on 2 July 2022. All subjects provided written informed consent. Informed consent was obtained from participants’ voluntary and confidential responses. Their participation in this study was completely voluntary and anonymous, and their refusal to participate would have no impact on their work or personal lives in the long run. All data was kept secure and confidential, and only the research team was allowed access to them in order to ensure their integrity. Within a two-week period, the data was collected online and in person from a simple random sample of different employees working in hospitals of Lahore (Pakistan) at different levels of responsibility. As a first step, we explained what the study was about as well as how we would protect confidentiality. There was a short survey available for them to complete that contained multiple items, including demographic data. The survey was available to all healthcare professionals who were officially employed by the hospital. After their eligibility for the study was confirmed, they were finally asked to complete a composited survey consisting of all the measures to be analyzed in the study.

## 5. Statistical Analysis

All statistical analysis was conducted with statistical software. Before hypothesis testing, we calculated descriptive statistics and inter-factor correlations for our sample to see the participants’ statistical characteristics. For the purpose of testing the mediating and moderating effects, a structural equation model was used.

### 5.1. Descriptive Statistics

Data were collected in person and online, and the total sample size was 350; among the participants, 245 (70%) were females and 105 (30%) were males. In terms of age, 35 (10%) were under 30 years, 175 (50%) were 30–39 years old, 105 (30%) were 40–49 years old, 24 (7%) were 50–59 years old, and the remaining participants were more than 60 years old. Similarly, there were 105 (30%) male participants and 245 (70%) female participants in the sample. There were 298 (85%) married participants, 49 (14%) were unmarried, and the remaining reported their relationship status as other. Similarly, we have collected data for different positions of the doctors, ranging from top to bottom: there are 35 (10%) consultants, 81 (23%) demonstrators, 109 (31%) registrars, 70 (20%) medical officers, and 56 (16%) post-graduate trainees. Similarly, we distributed them in terms of experience as well: 42 (12%) have three or less than three years of experience. There were 84 (24%) MHCPs with experience ranging from 3 to 6 years, and 122 (35%) who have experience ranging from 7 to 9 years. Similarly, there were 70 (20%) people with experience of 10 to 12 years, and 32 (9%) had more than 12 years. In Table 1, a summary of all the descriptive statistics is given, and Table 2 presents the mean and standard deviations of all the variables that were studied.

### 5.2. Results

From Table 3, we can see that age has a significant positive relation with position, experience, distress, health, innovation, and supervisor support, while age has a significant negative relation with eustress. When age increases, the level of eustress goes down. Eustress needs vitality, which decreases with the age. Similarly, the position has a significant positive relationship with experience, distress, innovative work behavior, and supervisor support. Position behaves similarly to age. As it is said, “great responsibilities come with great positions,” so the level of distress will be higher with higher positions. The position has an insignificant relationship with eustress and significant negative relation with health. Experience also has a significant positive relation with eustress and innovation. and supervisor support but has a significant negative relationship with distress and health. There is an insignificant relationship between eustress and distress but eustress and distress have significant positive and negative relationships respectively with the health, innovative behavior of an employee, and supervisor support. Innovative work behavior and supervisor supports are significantly positive related to each other.

Distress was found to have a significant negative relation with innovative work behavior (r=−0.29,p<0.003), and similarly, eustress has a significant positive relation with innovative work behavior (r=0.29,p<0.000) and employees’ innovative behavior (r=0.48,p<0.001).

### 5.3. Results of Exploratory Factor Analysis

All of the items were analyzed through Skewness and Kurtosis for data range. Before conducting the confirmatory factor analysis (CFA), the Exploratory Factor Analysis (EFA) was used to categorize the data set (see Table 4). The Kaiser–Meyer–Olkin (KMO) [26] and Bartlett’s test of sphericity [27] were used for sampling adequacy. The values were 0.833 and 4232.559, respectively, at *p* < 0.000 level. Researchers [28] ranged values for KMO from 0 to 1, with 0.6 suggested as a minimum value for good factor analysis.

### 5.4. Confirmatory Factor Analysis

**Figure 2:**
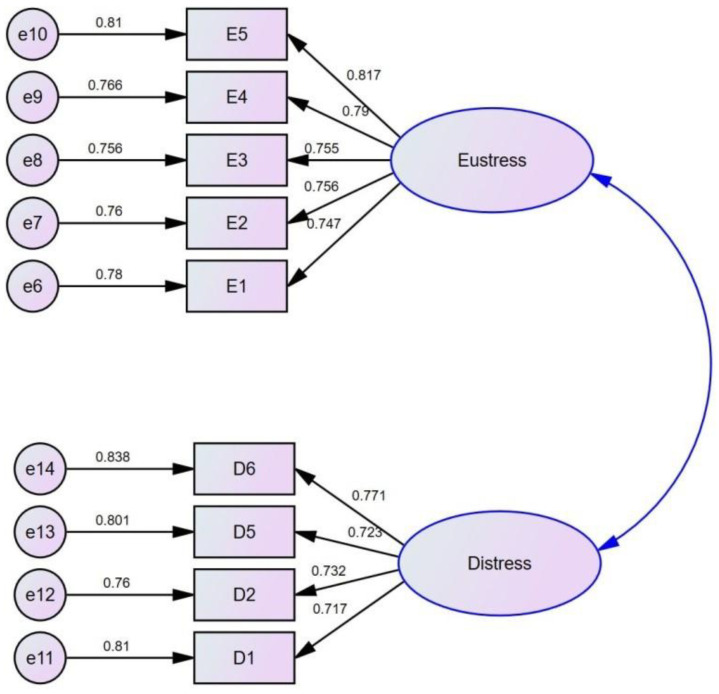
Confirmatory factor analysis, e6 to e14 are error terms, E1 to E5 are items of Eustress, and D1, D2, D5, and D6 are items of Distress, and Arrows are representing factor loadings.

The convergent and discriminant validity of each variable has been analyzed. We can see the convergent validity of all the variables since we have modified all the pre-developed questionnaires, so it was quite necessary to maintain the convergent validity of all the variables. It is observed that all factor loadings, construct validity, and average variance extracted (AVE) were more than 0.7, 0.7, and 0.5, respectively. Hence, we can claim that CFA meets the standards of convergent validity (see Figure 2).

Literature suggests that the AVE of all the variables should be bigger than maximum shared the square variance (MSV) and average shared square variance (ASV) [29], which can be seen in our case (see Table 5). The correlation of each construct was less than the square root of AVE. The goodness of model fit can be seen in Table 6, which presents the goodness of fit of the five variables.

### 5.5. Mediation Analysis

Through the structural equation modeling technique (SEM), the direct and indirect relations have been observed, which are presented in Table 7.

Statistical analysis [30] was exercised to verify the mediating part of health between stress (eustress and distress) and innovative work behavior. The dimensions of stress were divided into two categories, i.e., eustress and distress, while innovative behavior was a dependent variable. According to Baron and Kenny, if there is complete mediation, then indirect paths should be statistically significant (independent variable to mediating variable and then mediating variable to the dependent variable). Still, the direct path should be insignificant, and then there is full mediation between independent and dependent variables. On the other hand, for partial mediation, all the paths should be significant, but the beta value of the direct path should be more than the indirect path.

**Figure 3:**
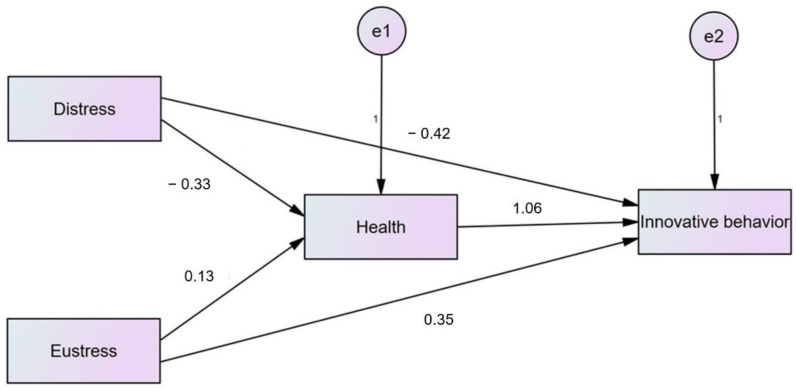
SEM analysis proposed model, e1 and e2 are error terms, and arrows are representing the beta values.

As shown in Figure 3, the impact of distress on health is quite significant. Similarly, the impact of health on innovation is quite significant. Still, the direct effect of distress on innovation is insignificant, which suggests, as per Barron and Kenny, that health fully mediates between distress and innovative work behavior, which supports our H3. However, on the other hand, eustress has a significant relation with health and innovative work behavior, but its beta value is smaller than the direct effect. Therefore, based on Barron and Kenny’s mediation analysis, health partially mediates innovative work behavior and eustress, so this result is not consistent with our H4.

### 5.6. Moderated Mediation Analysis

Through the structural equation modeling technique (SEM), the direct and indirect relations for moderation have been observed, which are presented in Table 8.

**Figure 4:**
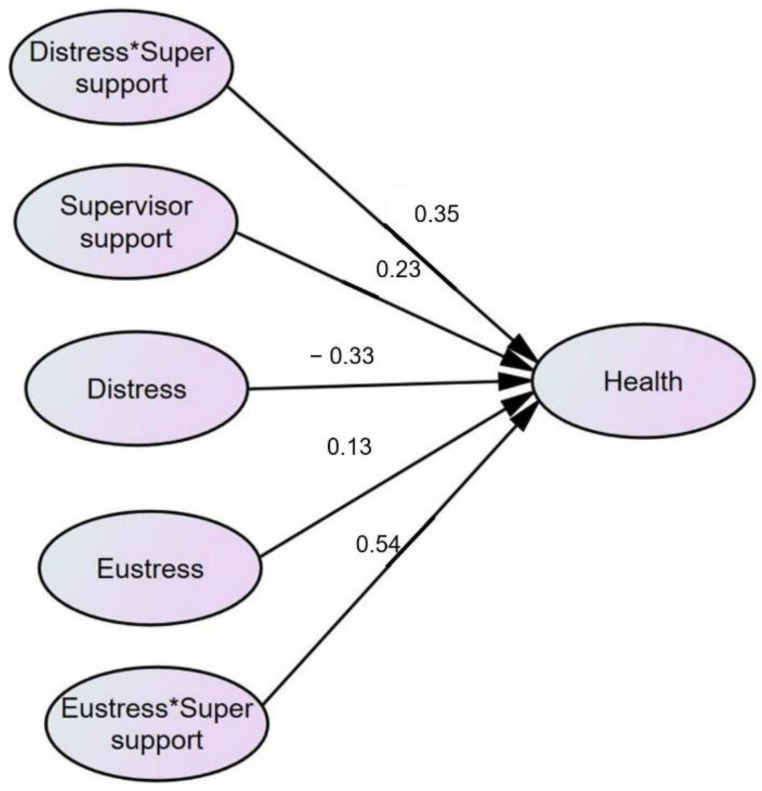
Moderation analysis supervisor support.

Statistical analysis [30] was exercised to verify the moderated mediation and mediated moderation analysis of the supervisor support among stress (Eustress and Distress), health, and innovative work behavior. The dimensions of stress were divided into two categories, i.e., eustress and distress, while innovative work behavior was a dependent variable. According to Baron and Kenny, if the effect of the independent variable on the dependent variable and the effect of the moderated variable on the dependent variable is significant and the effect of the interaction term is also significant, then moderation does exist. It can be seen from Table 8 that supervisor support is moderating between distress and health as a relationship between distress, supervisor support and health is significant, and the interaction term between supervisor support and distress also has a significant relationship with health, which states that supervisor support moderates’ distress and health (H5 is supported). Meanwhile, the interaction term between eustress and supervisor support does not have a significant relation with health, so supervisor support is not moderating between eustress and health (H6 is not supported). Furthermore, it is observed that our moderating variable also moderates eustress, distress, and innovative work behavior. The results show that this relation is also significant (see Figure 4).

### 5.7. Mediated Moderation Analysis

After performing the moderated mediation, we analyzed the moderated behavior of supervisor support between health and innovative work behavior.

From Table 9 given below, we can see that there is a significant relationship between health and innovation and also a significant relationship between supervisor support and the innovative work behavior of an employee. In addition, the interaction term (health × supervisor support) has a significant relation with innovative work behavior, so we can claim that supervisor support moderated health and innovative work behavior (H7 is supported).

**Figure 5:**
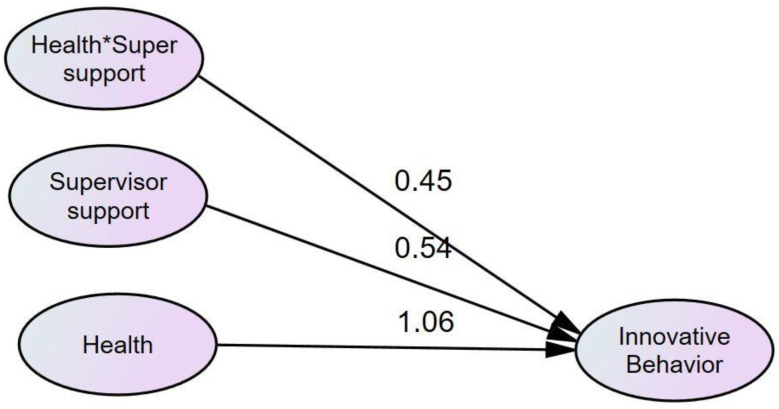
Moderation analysis of supervisor support with health and innovative work behavior.

Apart from that, it can be seen that this is positively moderated as the beta weight is 0.45, when supervisor support increases, it will positively affect the relationship between health and innovative work behavior (see Figure 5).

## 6. Discussion

In light of the growing prevalence of stress and the evidence of its detrimental effects on both employee and work-related outcomes, it is important to develop and test theoretical models to explain the phenomenon. In this study, we shed light on the mediating, moderating, and boundary conditions that influence the effects of stress on employee innovative behavior, a work outcome that has received limited attention in the literature on eustress despite its importance for innovative performance and competition. In line with our predictions, stress negatively affected innovative work behavior indirectly by reducing employees’ health. We argued the associations between stress and innovative behavior of the employee with mediating, moderating role of health and supervisor support respectively. The research concept was founded on the stress model of Lazarus and Folkman joined with the social support theory. An investigation was performed on data collected from 350 employees from different hospitals of Lahore, Pakistan. The inquiry verdicts showed the positive effects of eustress on individuals’ innovative behavior. These findings are in strong support of H2, clearly indicating that the eustress would stimulate the individual to feel happy or motivated and to achieve the desired goal with full energy and take challenges positively and consider them as a chance for self-grooming, learning, and increasing expertise, which helps to develop innovative behavior in the employees. On the other hand, distress has negative effects on innovative behavior, while there is a large body of evidence on distress and its effects on human health and on innovative work behavior, which supports our H1.

Furthermore, supervisor support does not moderate between eustress and health. Hence, H6 is not supported. Health partially mediates between eustress and innovative work behavior and supervisor support does not moderate between eustress and health. One possible reason for this partial mediation could be that since eustress has a direct significant relationship with innovative work behavior and eustress does not affect health negatively or positively, when people are happy and energetic, motivated, ready to face the challenges, and consider them as a part of training which will help them to grow professionally, they do not care about health and place primary focus on their work. In this case, supervisor support may have to some degree of positive impact and can enhance their performance further, but according to our findings, there is no need for supervisor support. One possible reason for this result could be that Pakistan is a country with limited resources and the health budget is not big. There are not enough hospitals as compared to the population. Additionally, Pakistan is one of the largest producers of medical healthcare professionals and is also the second largest exporter of medical healthcare professionals [2], as it is very difficult to obtain a job in hospitals. Once a person obtains a chance to work in the hospital as a medical healthcare professional, his energy level increases and he does not care about health but always focuses on his performance, which helps to bring innovation. One possible reason could be that it works in conjunction with another variable such as supervisor support. Clearly, high supervisor support will alleviate the impact of health in either way. So, health does not mediate between eustress and innovative work behavior.

Additionally, supervisor support appears to moderate distress and health. Hence, our H5 is supported. The study findings indicate that supervisors’ support for their employees influences their psychological well-being and job satisfaction. This study found that supervisor support directly influenced employees’ mental health in a positive and significant way. According to several studies, employees’ psychological well-being was positively affected by the support they received from their supervisors [31,32,33]. Finding this expected outcome might be a result of a supportive supervisor who offers guidance, assistance, and feedback to employees regarding complex situations that can arise at work, thereby alleviating stress impacting their psychological health and work performance [34]. In addition, managers can enhance psychological well-being by making their workers feel appreciated, respected, and supported. In addition to enhancing the innovative spirit of MHCPs in Pakistan, when you are supported by your supervisor, it increases trust and confidence in the sector.

Health and innovative work behavior moderate supervisor support; hence, H7 is supported. This moderation could be for several reasons. Subordinate support refers to how much supervisors are perceived by their subordinates as supportive, which is dependent on how favorable their immediate context is that impacts their personal outcomes [25,34]. Subordinates with supportive supervisors are praised and rewarded for effort exertion and good performance, react favorably to honest errors and find their work stimulating and meaningful. Supportive supervisors are concerned about their employees’ feelings and needs, value their efforts and contributions, and encourage them to feel self-determined, which in turn motivates them.

### Theoretical Contributions

This study contributes to the literature in several ways. Our study extends our current understanding of how stress impairs employees’ innovative work behaviors. The effects of stress on innovative work behavior have been largely revealed by research, but the mechanisms underlying such effects remain largely unknown. It is, however, essential to adopt a ‘process lens’ to understand the core psychological reactions that must be safeguarded to ensure that employees can continue to function effectively in stressful situations. In this study, we provide evidence to explain how stress affects innovative work behavior by investigating the mediating role of health and moderating role of supervisor support in MHCPs in Pakistan. We, therefore, extend the body of knowledge on how stressful conditions affect innovative behavior.

Our study contributes a key insight by identifying that supervisor support can buffer employees against the negative effects of stress and help them stay psychologically healthy. Even when the job is perceived as insecure, high supervisor support can help employees retain a passion for their work. As a result, they can maintain their motivation and, consequently, invest their energy in executing innovative behaviors. To date, working conditions have tended to focus on improving employee intrinsic motivation and, ultimately, innovative behaviors

Liu [35] ignored conditions for fostering employee motivation and innovation when unfavorable conditions prevail. In this study, we demonstrate that supervisor support can protect employees exposed to adverse workplace conditions, such as job insecurity, high workload due to COVID-19, and working with patients who are in danger of catching the disease. The findings from our research on supervisor support, health, and innovation in hospitals relate to our findings on moderated mediation and mediated moderation. Scholars have called for more research into the impact of supervisor support on creativity and innovation since they are not well studied. In the context of Pakistani MHCPs, our study extends the prior research on supervisor support and innovation to show that, in addition to improving health, supervisor support can also promote innovation.

## 7. Conclusions

### 7.1. Key Findings

This study examines the impact of distress and eustress on the innovative work behavior of an employee. Furthermore, a mediating role of health and moderation has been observed by the supervisor support analysis. In general perception and in different studies, it has been observed that if a supervisor is supportive, then the innovative work behavior of the employee will be enhanced, but in our study, supervisor support did not mediate between eustress and health. Eustress had a significant positive impact on innovation even without supervisor support. So, we can conclude that eustress is very important in any organization for innovative work behavior.

Our key findings support our hypotheses quite well. When supervisor support does not moderate between eustress and health, there is a partial mediation of health between eustress and innovative work behavior. There is also a negative correlation between distress and innovative behavior in the results of the study. Furthermore, health is a full mediator between distress and innovative work behavior. As a result, distress amplifies the negative effects of stress on health. However, supervisor support reduces the negative impact of distress on health, and the relationship between health and innovative work behavior improves as a result.

### 7.2. Practical Significance

The practical implications of the current study’s findings on stress are similar to those proposed by Cavanaugh et al. [23]. Hospitals and healthcare environments that want to improve employee happiness and motivation while reducing stress should focus on removing as much of the stress that these employees suffer as possible through the intervention of the supervisor. The supervisor should adjust organizational policies to accommodate this trend by providing employees with a clear awareness of what is expected of them and providing opportunities for advancement based on their performance. The results on eustress, on the other hand, are mixed. Healthcare employment is already demanding, but possible benefits from this form of stress were only discovered when the impediment of stress was eliminated. The presence of eustress exacerbated the deleterious consequences of distress. That is to say, unless we are certain that distress has been eliminated, eustress may do more harm than good, particularly for healthcare workers who are particularly susceptible to bad health. In this instance, it is also a good idea to keep distress to a minimum.

### 7.3. Limitations and Future Research

This study has a few limitations. To begin with, the study relies entirely on self-reports. People may not convey a true picture of themselves intentionally, or they may not be able to capture it accurately. The issue of the respondents’ exaggeration or expectations was brought up several times during the preceding discussion. Due to the lack of an experimental design, causal conclusions are impossible. There is uncertainty as to whether these effects are a result of distress or eustress. The likelihood of feeling stressed at work is higher for dissatisfied and unmotivated workers, for instance. There may also be a problem with a third variable that was not considered throughout the research. An unanticipated relationship may have been caused by a third variable influencing both variables. In addition, since the sample employed in this study was made up of healthcare workers, the findings are not generalizable. This means the results cannot be applied to other occupations based on the current sample.

Since Pakistan is a big country in size, it is tough to extrapolate the results to the entire country. We were only able to collect data from Lahore because it was challenging to contact medical healthcare providers during these unprecedented pandemic circumstances.

## Figures and Tables

**Table 1:** Demographic variables.

Variables		Number
Gender	Male	105 (30%)
	Female	245 (70%)
Age	<30	35 (10%)
	30–39	175 (50%)
	40–49	105 (30%)
	50–59	24 (7%)
	≥60	11 (3%)
Marital Status	Married	298 (85%)
	Unmarried	49 (14%)
	Others	3 (1%)
Position	Consultants	35 (10%)
	Demonstrators	81 (23%)
	Registrars	109 (31%)
	Medical Officers	70 (20%)
	Postgraduate trainees	56 (16%)
Experience	0–3 years	42 (12%)
	4–6 years	84 (24%)
	7–9 years	122 (35%)
	10–12 years	70 (20%)
	More than 12 years	32 (9%)

**Table 2:** Mean and standard deviations of the studied variables.

Variables	Mean	Standard Deviation
Eustress	3.45	0.85
Distress	2.89	1.05
Health	3.32	0.93
S. Support	3.65	0.87
Inn. Work Behavior	3.55	1.15

**Table 3:** Correlations among the studied variables.

Variable	Age	Position	Experience	Eustress	Distress	Innovation
Age	-	-	-	-	-	-
Position	0.45 **	-	-	-	-	-
Experience	0.40 **	0.45 **	-	-	-	-
Eustress	−0.23 **	0.15	0.32 **	-	-	-
Distress	0.43 *	0.21 **	−0.4 **	0.21	-	-
Health	0.32 **	−0.30 *	−0.32 **	0.32 **	−0.31 *	-
Inn. Work Behavior	0.23 **	0.19 **	0.41**	0.48 **	−0.29 **	-
S. Support	0.15 **	0.23 **	0.12	0.32 **	−045 **	0.49 **

* Significant at 0.01 < *p* < 0.05; ** Significant at 0.001 < *p* < 0.01.

**Table 4:** Factor Loadings, Pattern Matrix, and Communalities.

Items	Factor’s Loadings						Communalities
Eustress1	0.747	0.863					0.735
Eustress2	0.756	0.842					0.743
Eustress3	0.755	0.795					0.756
Eustress4	0.790	0.779					0.766
Eustress5	0.817	0.795					0.800
Distress1	0.717		0.764				0.815
Distress2	0.732		0.770				0.760
Distress5	0.723		0.783				0.801
Distress6	0.771		0.931				0.838
Health1	0.752			0.865			0.812
Health2	0.805			0.765			0.845
Health4	0.811			0.832			0.786
Health6	0.829			0.754			0.851
Health7	0.788			0.897			0.864
Health8	0.850			0.812			0.977
S. Support.1	0.882				0.794		0.683
S. Support.3	0.872				0.944		0.894
S. Support.4	0.910				0.881		0.882
S. Support.5	0.735				0.824		0.849
S. Support.6	0.813				0.812		0.796
S. Support.8	0.965				0.897		0.856
Inn. Work Behavior.2	0.766					0.826	0.718
Inn. Work Behavior.5	0.826					0.783	0.808
Inn. Work Behavior.6	0.823					0.801	0.788
Inn. Work Behavior.7	0.782					0.788	0.775
Inn. Work Behavior.8	0.710					0.819	0.729
Inn. Work Behavior.3	0.804					0.806	0.786
Inn.Work Behavior.14	0.831					0.842	0.803
Inn.Work Behavior.15	0.711					0.819	0.656
Inn.Work Behavior.16	0.791					0.864	0.839
Inn.Work Behavior.17	0.728					0.916	0.769
Inn.Work Behavior.18	0.811					0.827	0.577

**Table 5:** Average variance extracted, Composite reliability, and collective Cronbach Alpha.

Variables	Measurement Items	Standard Loadings	AVE	CR	Cronbach Alpha
Eustress	Eustress1	0.863	0.78	0.802	0.91
	Eustress2	0.842			
	Eustress3	0.795			
	Eustress4	0.779			
	Eustress5	0.795			
Distress	Distress1	0.717	0.69	0.75	0.95
	Distress2	0.732			
	Distress5	0.723			
	Distress6	0.771			
Health	Health1	0.882	0.73	0.81	0.906
	Health2	0.872			
	Health4	0.910			
	Health6	0.735			
	Health7	0.813			
	Health8	0.912			
Innovation	Inn. Work Behavior.2	0.766	0.71	0.83	0.93
	Inn. Work Behavior.5	0.826			
	Inn. Work Behavior.6	0.823			
	Inn. Work Behavior.7	0.782			
	Inn. Work Behavior.8	0.710			
	Inn. Work Behavior.3	0.804			
	Inn. Work Behavior.14	0.831			
	Inn. Work Behavior.15	0.711			
	Inn. Work Behavior.16	0.791			
	Inn. Work Behavior.17	0.728			
	Inn. Work Behavior.18	0.811			
Supervisor Support	S.Support.1	0.766	0.81	0.85	0.941
	S.Support.2	0.826			
	S.Support.3	0.823			
	S.Support.4	0.782			
	S.Support.5	0.710			
	S.Support.6	0.804			
	S.Support. 8	0.831			

**Table 6:** Model fitness.

	Direct Effect	Indirect Effect
GFI	0.93	0.96
AGFI	0.97	0.90
TLI	0.89	0.98
CFI	0.93	0.88
RMSEA	0.054	0.035

**Table 7:** Hypotheses testing.

Hypothesis Tested	Relations	Beta Coefficients	*p*-Value	Remarks
	Eustress → Health	0.13	0.000	Significant
	Distress → Health	−0.33	0.000	Significant
	Eustress→Inn.Work Behavior	0.35	0.004	Significant
	Distress →Inn.Work Behavior	−0.42	0.099	Insignificant
	Health → Inn.Work Behavior	1.06	0.000	Significant

**Table 8:** Direct and indirect relations of studies variables.

Hypothesis Tested	Relations	β Coefficients	*p*-Value	Remarks
	Eustress → Health	0.13	0.000	Significant
	S.Support → Health	0.23	0.024	Significant
	Eustress × S.Support → Health	0.54	0.094	Insignificant
	Eustress × S.Support → Inn.	0.49	0.000	Significant
	Distress → Health	−0.33	0.000	Significant
	Distress × S.Support → Health	0.35	0.004	Significant
	Distress × S.Support → Inn.	0.65	0.000	Significant

**Table 9:** Moderated behavior of supervisor support between health and innovative work behavior.

Hypothesis Tested	Relations	Beta (β) Coefficients	*p*-Value	Remarks
	Health → Inn.	1.06	0.000	Significant
	S.Support → Inn.	0.54	0.000	Significant
	Health × S.Support → Inn.	0.45	0.000	Significant

## Data Availability

The data presented in this study are available on request from the corresponding author.
